# Isolation and characterization of two *Acinetobacter* species able to degrade 3-methylindole

**DOI:** 10.1371/journal.pone.0211275

**Published:** 2019-01-28

**Authors:** Tujuba Ayele Tesso, Aijuan Zheng, Huiyi Cai, Guohua Liu

**Affiliations:** 1 The Key Laboratory of Feed Biotechnology of Ministry of Agriculture, National Engineering Research Center of Biological Feed, Feed Research Institute, Chinese Academy of Agricultural Sciences, Beijing, China; 2 Department of Biology, Faculty of Natural Sciences, Mettu University, Mettu, Ethiopia; Babasaheb Bhimrao Ambedkar University, INDIA

## Abstract

3-Methylindole (3MI) or Skatole is a volatile lipophilic organic compound produced by anoxic metabolism of L-tryptophan and associated with animal farming and industrial processing wastes. Pure cultures of bacteria capable of utilizing 3MI were isolated from chicken manure using enrichment culture techniques. The bacteria were identified as *Acinetobacter toweneri* NTA1-2A and *Acinetobacte*r *guillouiae* TAT1-6A, based on 16S rDNA gene amplicon sequence data. The optimal temperature and pH for degradation of 3MI were established using single factor experiments. Strain tolerance was assessed over a range of initial concentrations of 3MI, and the effects of initial concentration on subsequent microbial 3MI degradation were also measured. During the degradation experiment, concentrations of 3MI were quantified by reverse-phase high-performance liquid chromatography (HPLC). The strains were capable of degrade initial concentrations of 3MI ranging from 65–200 mg/L. The degradation efficiency was >85% in 6 days for both strains when the initial concentration is less than 200 mg/L. The strains were tested for enzymatic activity using 65 mg/L 3MI. The enzyme extracts of NTA1-2A and TAT1-6A from the 3MI medium degraded 71.46% and 60.71% of 3MI respectively, but no appreciable change in 3MI concentration in the control group was witnessed. Our experiment revealed betaine and choline were identified as 3MI degradation metabolites by both strains while nitroso-pyrrolidine and beta-alaninebetaine formed by NTA1-2A and TAT1-6A strains respectively. The NTA1-2A and TAT1-6A strains removed 84.32% and 81.39% 3MI respectively from chicken manure during fermentation in 8 days and showed a statistically significant difference (*P <* 0.05) compared with the control group. The optimum temperature and pH were 31°C and 6 respectively, for 3MI degradation by *A*. *toweneri* NTA1-2A and *A*. *guillouiae* TAT1-6A. We concluded that *A*. *toweneri* NTA1-2A and *A*. *guillouiae* TAT1-6A are potential strains of interest to degrade 3MI and control odorant in poultry and other livestock industries.

## Introduction

3-Methylindole (3MI) is a common metabolite found in sewage and mammalian and avian feces, and is a well-known, foul-smelling fecal odorant [1–3]. The production and appearance of this compound has practical consequences for farm animal production, human health and environmental pollution [4, 5]. It is the third most harmful odorant in livestock farming, following ammonia and H_2_S. In high concentrations, 3MI not only pollutes the air in barns and around the farm, but also increases the risk of boar taint owing to animal’s absorption of 3MI from the air. Moreover, due to their methyl derivatives, 3MI and other indolic compounds have mutagenic properties [6]. For instance, 3MI has been reported to cause severe pulmonary edema, emphysema, lung disease [7], hemoglobinuria, and hemolysis [8] in livestock such as cattle and goats. Due to the increasing demands of livestock production, 3MI is an environmental and public health issue contributing to the release of noxious odors on animal farms [5].

Due to their persistence, mobility, and potential impacts on the environment and human and animal welfare, studies of indolic compounds (such as 3MI) from waste treatment plants have highlighted microorganisms capable of its biodegradation [9]. For example, several studies have investigated bacterial and fungal biodegradation of indolic compounds [9–11] under variable conditions. In a study of pig and chicken manure composting processes, Kohda *et al*. [2] isolated three species of 3MI-degrading *Clostridium* (*C*. *aminovalericum*, *C*. *carnis*, and *C*. *malenominatum*). In another study, researchers isolated a pure culture of *Pseudomonas aeruginosa* capable of utilizing 262.34 mg/L 3MI completely in 24 days using enrichment cultures [12]. The degradation of 3MI was also reported in a methanogenic consortium isolated from an enrichment of wetland soil [13]. *Lactobacillus brevis* 1.12 was reported best out of four lactic acid bacteria in its tolerance and ability of removing 3-methylindole [14]. Biotransformation of 3MI was also recorded in methanogenic consortia with complete mineralization by sulfate reducing bacteria derived from marine sediment of Victoria Harbor [15]. A new 3-methylindole-degrading purple non-sulfur bacterium, *Rhodopseudomonas palustris* WKU-KDNS3, was isolated from a swine waste lagoon using an enrichment technique [16] No studies to date have noted the ability to degrade 3MI in *Acinetobacter* species, although some species of the genus *Acinetobacter* have been reported to degrade n-alkane and long chain hydrocarbons [17, 18] and aromatic compounds such as phenol and chlorophenol [19–21]. Additionally, despite its ubiquity, use of *Acinetobacter* for environmental remediation is under-reported. Here, we present the first report of 3MI degradation in two *Acinetobacter* strains isolated from chicken manure. The strains were also tested for their ability to remove 3MI from chicken manure and reduce the foul-smelling odorant.

The objectives of this study were to evaluate the 3MI degradation capacity of *Acinetobacter toweneri* NTA1-2A and *Acinetobacte*r *guillouiae* TAT1-6A and analyze performance-related factors such as temperature, pH, and 3MI initial concentrations. This work offers a baseline for further investigations to address environmental pollution emanating from indolic and other nitrogenous pollutants in the rapidly expanding poultry and livestock industries.

## Materials and methods

### Isolation of 3MI-degrading bacteria

A minimal salt medium (MSM) containing 150 mL of a solution (per L of distilled water) of 0.80 g K_2_HPO_4_, 0.20 g KH_2_PO_4_, 0.05 g CaCl_2_, 0.5 g MgCl_2_, 0.01 g FeCl_2_, 1.0 g (NH_4_)_2_SO_4_, 5.0 g NaCl, and 1.0 g yeast extract (Difco, Detroit, MI, USA), amended with 3MI in a 250-mL Erlenmeyer flask was prepared for the enrichment and isolation of aerobic 3MI-degrading bacteria [22]. A saline solution (0.9% NaCl) was also prepared. The saline and MSM solutions were autoclaved for 20 min at 121°C. A 250-mL Erlenmeyer flask containing 150 mL sterile saline was inoculated with 10 g of chicken manure from a breeding facility at the Chinese Academy of Agricultural Sciences (CAAS, Beijing, China; with the permission of and witnessed by the staff–in-charge of the breeding facility) and oscillated on an incubator shaker at 140 rotations per min (rpm) for 30 min. After 20 min of settling, 1 mL of the supernatant was transferred to MSM amended with 0.5 mM of 3MI and cultured at 30°C for 2 d. One mL of the above culture was transferred to a sterile MSM and cultured for 2 d at the same temperature. This procedure was repeated 10 times. The pH was adjusted to 7.0 using a 1 N solution of NaOH and HCl before sterilization. Pre-sterilized (at 121°C for 20 min) MSM was amended with 65.58 mg/L (0.5 mM) of 3MI dissolved in hot, sterilized water (50 mL, 50°C) and filtered with 0.22-μm pore-sized PTFE membrane syringe filters (JIN TENG) to be used during the degradation experiment. The enrichment culture was started by transferring 1.0 mL inoculant to sterile MSM amended with 65.58 mg/L (0.5 mM) of 3MI, which was gradually increased. Colonies exhibiting different morphological characteristics were isolated and purified by serially streaking on MSM agar plates. These strains were activated using yeast extract (1 g/L) with MSM for 24 h. Then 5.0 mL of each strain solution were transferred to sterile centrifuge tubes and centrifuged for 10 min at 8000 rpm. The supernatant was discarded, and pellets were washed three times with sterilized saline. Pellets were re-suspended in sterilized saline. These suspensions were used to separately inoculate 150 mL each of MSM per strain, and then cultured using the shaking culture method at 30°C and pH 7 with shaking speed of 140 rpm for 48 h. After culturing, 3MI concentrations of all culture media were measured and compared. The strains with the highest efficiency of 3MI degradation were targeted.

### Identification of the selected strains NTA1-2A and TAT-6A

The selected strains (NTA1-2A and TAT-6A) were identified by morphological and physiological characteristics, as well as 16S rDNA gene amplicon sequence analysis. The strains were streaked on agar plates and cultured at 30°C for 36 h to observe morphological characteristics under a light microscope. Microbial biomass in culture flasks was determined by measuring absorbance or optical density (OD) at 600 nm wavelength with an UV-visible spectrophotometer (UV-1700 Spectrophotometer, Shimadzu, Kyoto, Japan). Microbial characteristics and colony morphology were observed under a compound light microscope. The physiological and biochemical characteristics of the pure isolates were assessed using Biolog GEN III MicroPlate (Biolog, Hayward, CA, USA).

The 16S rDNA region was amplified by PCR using the bacterial primer set 16SF-16SR (16SF 5’-TTGGAGAGTTTGATCCTGGCTC-3’; 16SR 5’-ACGTCATCCCCACCTTCCTC-3’) [23]. PCR products were purified and sequenced by Tsingke Biotechnology Beijing Co., (Beijing, China). PCR amplification was performed using the SECCO 2 × TsingKE Master Mix (Code No.: TSE003) for 10 min at 94°C, and cycling was performed as follows: 94°C for 10 s, 55°C for 10 s, and 72°C for 15 s for 30 cycles (Applied Biosystem 2720 Thermal Cycler, Foster city California, USA). Denaturation was carried out for 2 min at 96°C, and annealing and primer extension were performed at 96°C for 10 s, 50°C for 10 s, and 60°C for 3 min for 30 cycles. The sequences were compared with those of other microorganisms in GenBank database (http://www.ncbi.nlm.nih.gov) [24] using the online Basic Local Alignment Search Tool program (BLAST). The Phylogenetic tree was constructed using neighbor joining method [25] and the evolutionary distances were computed using the Kimura 2-parameter method [26]. Bootstrap replications (1000) were conducted in MEGA7 [27]

### Assessing 3MI degradation

A MSM amended with 65.58 mg/L (0.5 mM) was used to assess the degradation of 3MI by selected strains. To begin, 100 **μ**L of activated bacterial culture were used to inoculate 150 mL of MSM in a 250-mL Erlenmeyer flask amended with 3MI, and strains were incubated at 30°C with a shaking speed of 140 rpm over 24 h. Samples (2 mL) were collected every 24 h during the incubation period and analyzed for 3MI removal.

### Single factors affecting 3MI degradation by the strains

Two single factor experiments were conducted to study the characteristics of the strains NTA1-2A and TAT1-6A under different culture conditions, including pH and temperature (T). All operations were carried out under sterile conditions. For the pH factor, the initial pH was adjusted to 5, 6, 7, 8 and 9 respectively using 1 mol/L HCl or 1 mol/L NaOH solution at 30°C and 140 rpm shaking speed. For the temperature factor, the culture temperature was set to 25, 28, 31, 34 and 37°C separately with constant pH of 7. The initial concentration of 3MI remained constant during both factors.

### Effect of 3MI initial concentrations on 3MI degradation

Individual colonies of pure culture on Petri plates were selected to inoculate pre-sterilized MSM containing 1.0 g/L yeast extract (Difco, Detroit, MI) in Erlenmeyer flasks which were incubated in a shaking incubator at 140 rpm and 31°C for 24 h. The 3MI degradation experiment was started by inoculating 100 μL active cultures to pre-sterilized MSM amended with 3MI at concentrations 0.5, 1.0, 1.5, 2.0 and 2.5 mM which correspond to 65.58, 131.17, 196.75, 262.34 and 327.92 mg/L respectively (each in triplicate). Non-inoculated flasks with pre-sterilized MSM served as control.

### Enzyme activity test

The strains NTA1-2A and TAT1-6A were tested for enzymatic activity of 3MI degradation. The culture media of the strains were filtered, and the supernatant used to degrade 0.5 mM (65.58 mg/L) of 3MI for 48 h at pH 6 and 31°C. The concentration of 3MI was determined after incubation at 31°C for 48 h.

### Chicken manure fermentation experiment

Chicken manure was collected from the CAAS Poultry Breeding Farm and fermented for eight days using the two strains previously isolated with permission and witness of Animal Ethical Committee of Feed Research Institute, CAAS. In this experiment, 4 kg of chicken manure was added to 10 L buckets in triplicate and fermented for 8 days using NTA1-2A (10^6^ CFU/mL) and TAT1-6A (10^7^ CFU/mL), which was added to each sample and mixed well. Non-inoculated manure was used as a control for each treatment. Samples were collected before treatment and during the fermentation every 2 d and stored at −21°C in plastic bags. One gram of each sample was mixed with 3 mL of methanol, heated in a water bath for 20 min at 40°C and vortex mixed every 5 min. Finally, the sample was placed in the refrigerator at −21°C for 15 min to accelerate precipitation speed. The supernatant was carefully collected and prepared for HPLC analysis by centrifuging at 12000 rpm for 10 min, and filtering onto 0.22-μL pore-sized syringe filter membrane. The samples were stored at 4°C and analyzed using HPLC for 3MI concentrations using standard 3MI (Shanghai Macklin Biochemical Co., Ltd, Shanghai, China).

### Sample preparation for HPLC analysis

An Agilent C18 column (Agilent Technologies, Inc., Santa Clara, CA, USA), 250 mm x 4.6 mm, with a particle size of 5 μm was used for high performance liquid chromatography (HPLC) (SHIMADZU LC-15C, Kyoto, Japan). The mobile phase contained 1% acetic acid and methanol at a ratio of 50:50 (v:v). The flow rate, injection volume, detection wavelength and column temperature were 0.6 mL/min, 20 μL, 260 nm and 30°C, respectively. In preparation for HPLC analysis, 2 mL of culture samples were collected and prepared by centrifuging (8,000×g) (Eppendorf 5427 R Centrifuge, Eppendorf AG, Hamburg, Germany) and filtering through 0.22-μm pore-size PTFE membrane syringe filters. Quantities of the analytes were examined from a standard curve prepared using standard 3MI (Shanghai Macklin Biochemical Co., Ltd, Shanghai, China). The quantity of 3MI in each sample was determined in mg/kg. The recovered quantity was also calculated as the percentage value with respect to the total concentration of 3MI added in the medium.

### 3-methylindole degradation metabolites analysis

The LC-MS analysis was carried out using DIONEX Ultimate 3000 Ultra High Performance Liquid Chromatography (UHPLC) with Column: ACQUITY BEH C18 1.7μm, 2.1×50 mm; Liquid phase conditions: A: water (containing 2 mmoL / Lammonium formate and 0.1% formic acid), D: acetonitrile; Gradient elution, 0~18min analysis time, 5 μL injection volume, and flow rate 0.25 mL/min. Mass spectrometry parameters (Thermo Q-Exactive) with ion source ESI (±) full scan, secondary data dependent scan (Full MS/dd-MS^2^) were performed. During our experiment, the supernatant media solution of the two strains (NTA1-2A and TAT1-6A) treated with 3MI were added with 10 times methanol to precipitate the supernatant fluid of culture media. After centrifugation at 10,000 rpm for 10 min, 5 μL of the injection was taken for LC-MS/MS^2^ analysis. The LC / MS data were preconditioned using Compound Discovery software from Thermo to obtain the variables retention time, and mass-to-charge ratio (rt_mz), compound molecular weight (MW) of observations (samples), and peak intensities and imported data matrix into MetaboAnalyst3.5 for principal component analysis (PCA). Based on the information, the possible compounds were searched from online for secondary metabolites data base such as METLIN (https://metlin.scripps.edu) using their molecular weight.

### Statistical analysis

Results were presented as mean values with standard error for triplicate assays. Simple linear regressions were calculated using Microsoft Excel 2010 for 3MI standard solutions with different gradient concentrations and for the growth of *Acinetobacter* strains in the MSM with 3MI. The data were subjected to one-way analysis of variance (ANOVA) and independent T-tests. To analyze the difference between treatment groups, SPSS Version 20 (IBM Corp., Armonk, NY, USA) was used.

## Results

### Identification of Strains NTA1-2A and TAT1-6A

**Fig 1:**
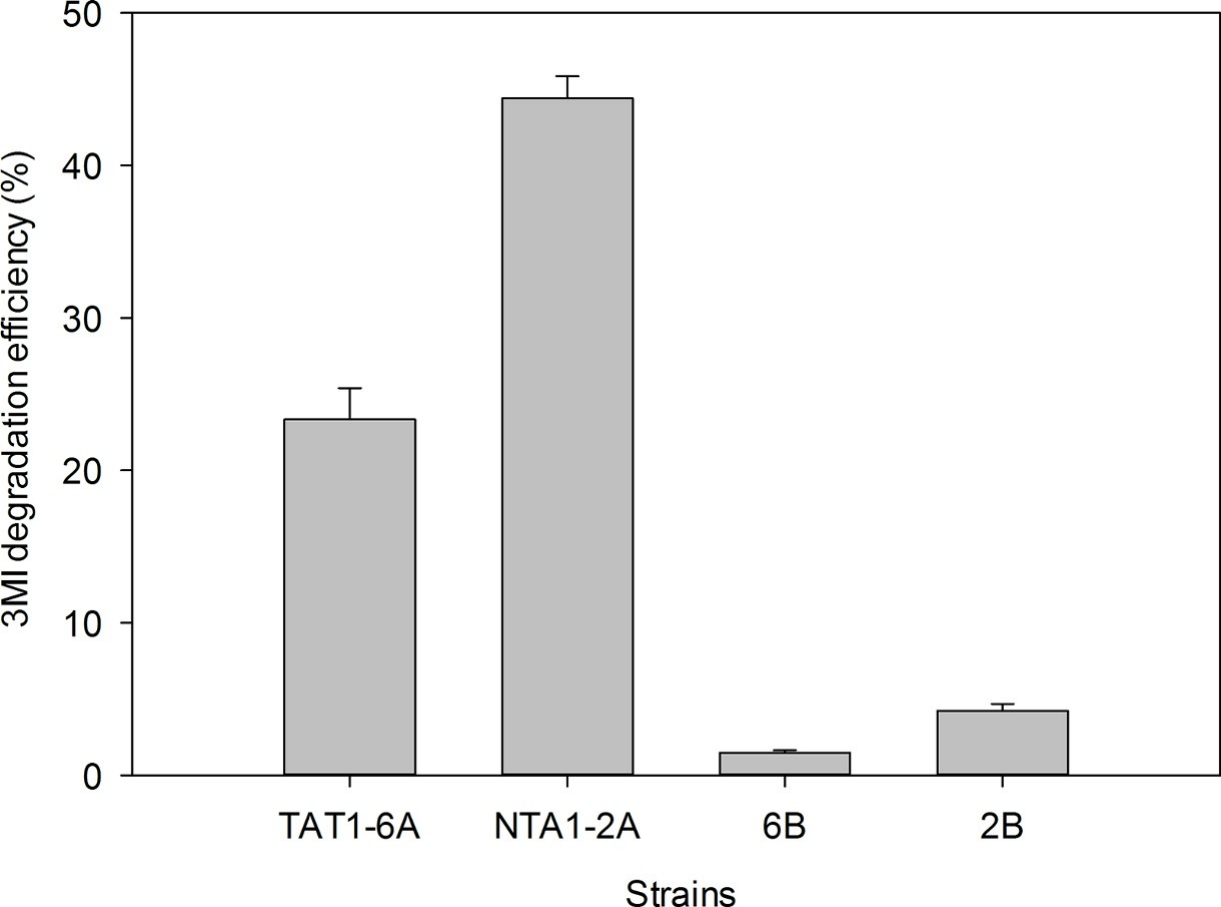
3MI degradation efficiency of the different strains from chicken manure.

Four colonies were observed on the enrichment culture medium. All four were tested for the ability to degrade 3MI (Fig 1). Of these, strains NTA1-2A and TAT1-6A degraded the most 3MI over a short period of time.

Colony morphology of the strains was observed under a compound light microscope with high magnification power. The strains were aerobic, Gram-negative, non-motile, rod-shaped and occurred in a pair or in chains when observed under a compound light microscope. Physiological and biochemical test results revealed that both strains utilize L-glutamic acid, p-Hydroxy-phenylacetic acid, methyl pyruvate, L-lactic acid, α-Keto-glutaric acid, L-malic acid, Tween 40, α-hydroxy-butyric acid, β-hydroxy-D, L-butyric acid, α-keto-butyric acid, propionic acid, and acetic acid as carbon sources, while dextrin and bromo-succinic acid were only utilized by strain TAT1-6A. Neither strain utilized mannose, D-fructose, D-sorbitol, D-mannitol, L-alanine, L-arginine, α-D-glucose, D-maltose or L-aspartic acid (Table 1). The strains exhibited no chemical sensitivity to rifamycin SV, lincomycin, guanidine HCl, Niaproof-4, vancomycin, tetraz blue, or potassium tellurite. Strain NTA1-2A was sensitive to tetrazolium violet, aztreonam, and sodium butyrate but TAT1-6A tested positive for growth, indicating no sensitivities to these compounds (Table 2).

**Table 1:** Biochemical and physiological characteristics of NTA1-2A and TAT1-6A strains using the GEN III MicroPlate identification tool.

Carbon source	Strain Reaction	
	TAT1-6A	NTA1-2A
Dextrin	+	*−*
L-glutamic Acid	+	+
p-Hydroxy-phenylacetic acid	+	+
Methyl pyruvate	+	+
D-fructose	*−*	*−*
α-D-glucose	*−*	*−*
L-Lactic acid	+	+
L-aspartic acid	*−*	*−*
α-Keto-glutaric acid	+	+
D-mannitol	*−*	*−*
D-maltose	*−*	*−*
L-Malic acid	+	+
Bromo-Succinic acid	+	*−*
D-sorbitol	*−*	*−*
Tween 40	+	+
α-hydroxy-Butyric acid	+	+
β-hydroxy-D, L-Butyric acid	+	+
α-keto-butyric acid	+	+
Propionic acid	+	+
Acetic acid	+	+
Mannose	*−*	*−*
L-alanine	*−*	*−*
L-arginine	*−*	*−*

**Table 2:** Chemical sensitivity of strains NTA1-2A and TAT1-6A strains using GEN III MicroPlate identification tool.

Chemicals	Strains Reaction	
	TAT1-6A	NTA1-2A
D-Serine	+	w^+^
Rifamycin SV	+	+
Lincomycin	+	+
Guanidine HCl	+	+
Niaproof-4	+	+
Vancomycin	+	+
Tetrazolium Violet	+	*−*
Tetraz blue	+	+
Potassium Tellurite	+	+
Aztreonam	+	*−*
Sodium Butyrate	+	*−*
Sodium Bromate (W+)	w^+^	*−*
Nalidixic Acid	*−*	+
pH 5	*−*	*−*
pH 6	+	+
1% NaCl	+	+
4% NaCl	*−*	*−*
8% NaCl	*−*	*−*

^+^ Positive, ^−^ negative and, w^+^ weakly positive

**Fig 2:**
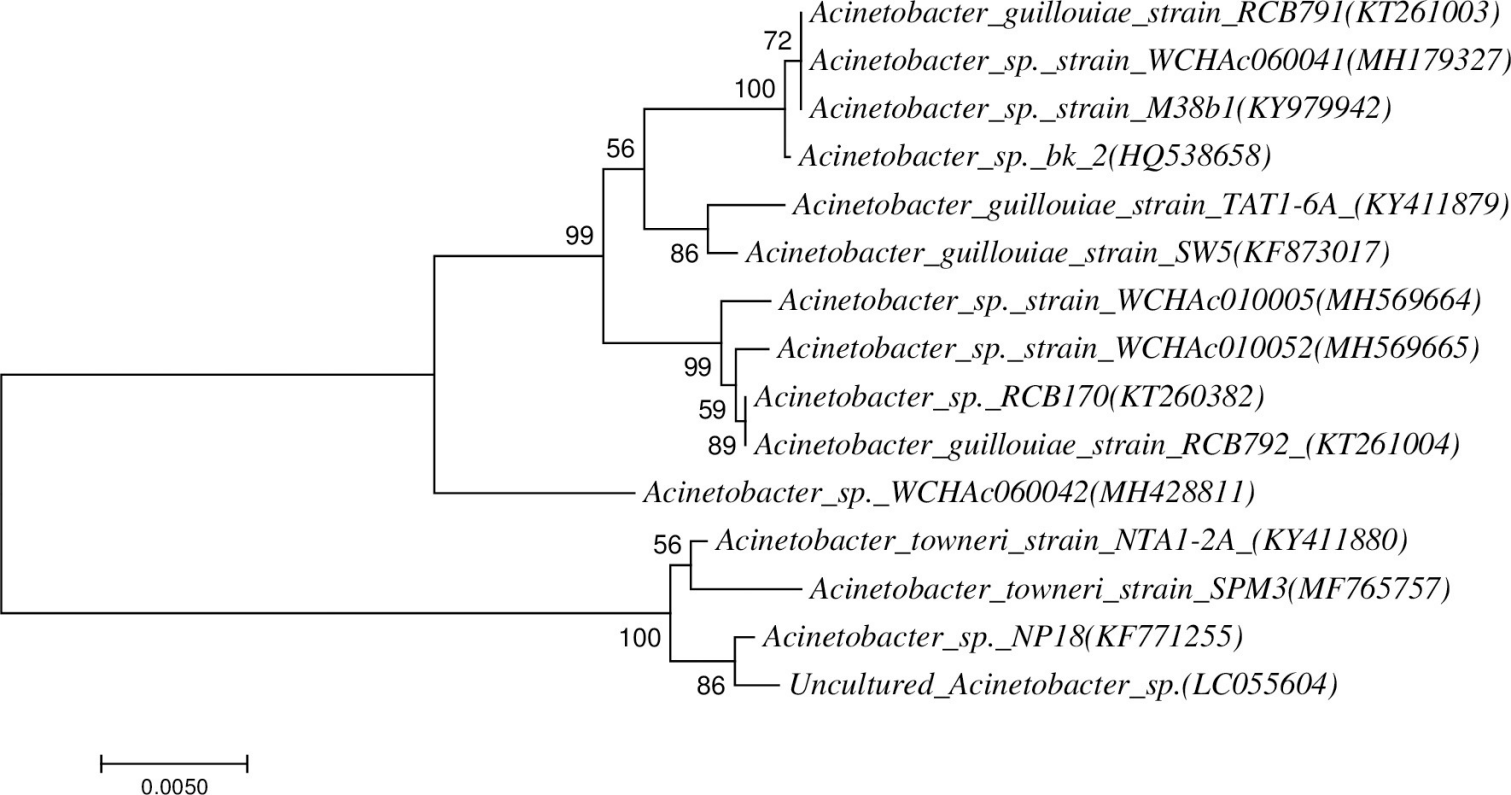
Neighbor joining tree of strains *Acinetobacter towneri* NTA1-2A and *Acinetobacter guillouiae* TAT1-6A.

The 16S rDNA gene amplicon products of DNA isolated from strains NTA1-2A and TAT1-6A (1407 bp and 1408 bp, respectively) were sequenced and showed 99% sequence similarity with *Acinetobacter towneri* and *Acinetobacter guillouiae* homologous genes in GenBank using BLAST. Combining these, our strains NTA1-2A and TAT1-6A identified as *Acinetobacter towneri* and *Acinetobacter guillouiae* on the basis of 16S rRNA gene sequencing. The 16S rRNA gene sequences of the strains NTA1-2A and TAT1-6A have been deposited in NCBI database under the GenBank accession numbers of KY411880 and KY411879. Phylogenetic analysis showed that strain NTA1-2A and TAT1-6A fell within other members of *Acinetobacter* near Acinetobacter *towneri_strain_SPM3* and *Acinetobacter guillouiae_strain_SW5* respectively (Fig 2). The strains have been named *Acinetobacter towneri* NTA1-2A and *Acinetobacter guillouiae* TAT1-6A. We preserved the strains in the China General Microbiological Culture Collection Center (CGMCC) under the preservation numbers CGMCC No. 14001 and 14002, respectively.

### Effect of temperature and pH on growth and 3MI degradation

**Fig 3:**
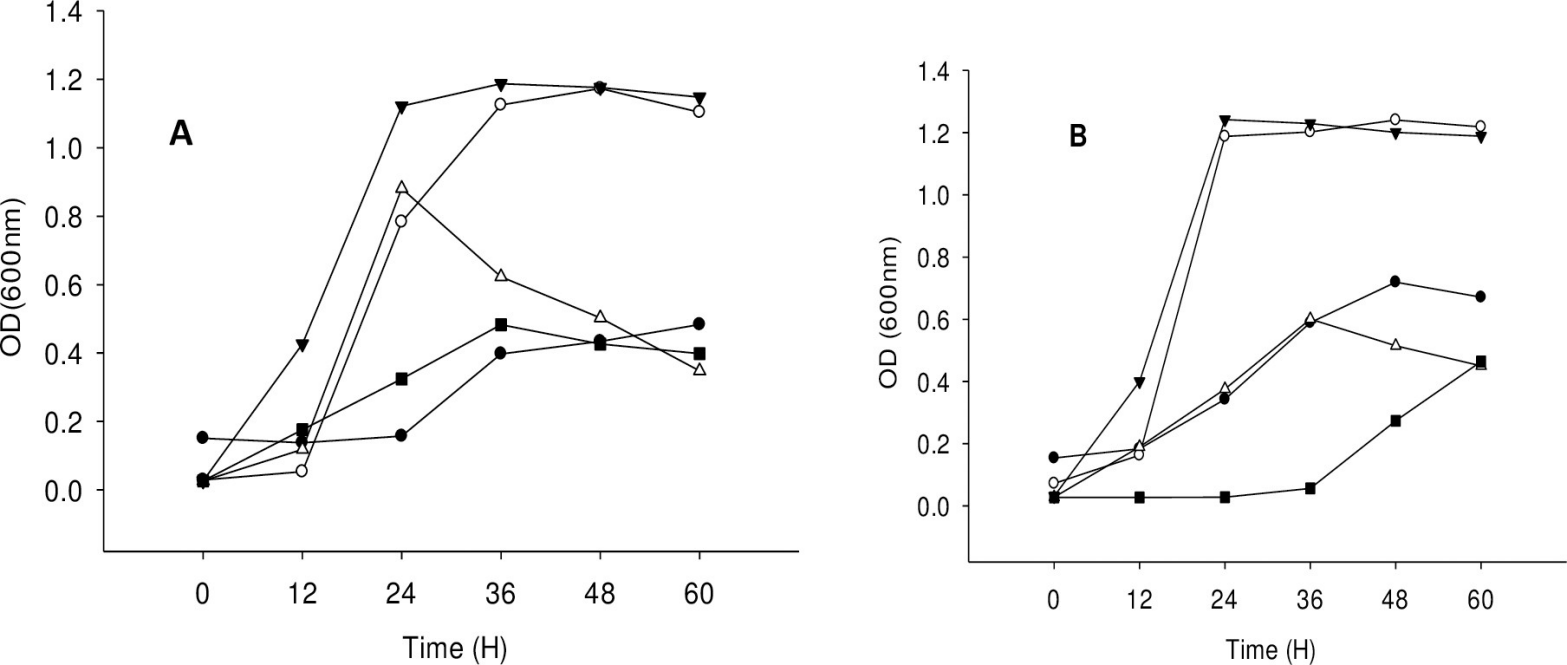
**Proliferation of the strains under different temperature; (A) *Acinetobacter toweneri* NTA1-2A, (B) *Acinetobacter guillouiae* TAT1-6A.** Symbols: solid circle, 25°C; open circle, 28°C; solid triangle, 31°C; open triangle, 34°C; solid square, 37°C at pH 6 and 1 mM of 3MI concentration.

**Fig 4:**
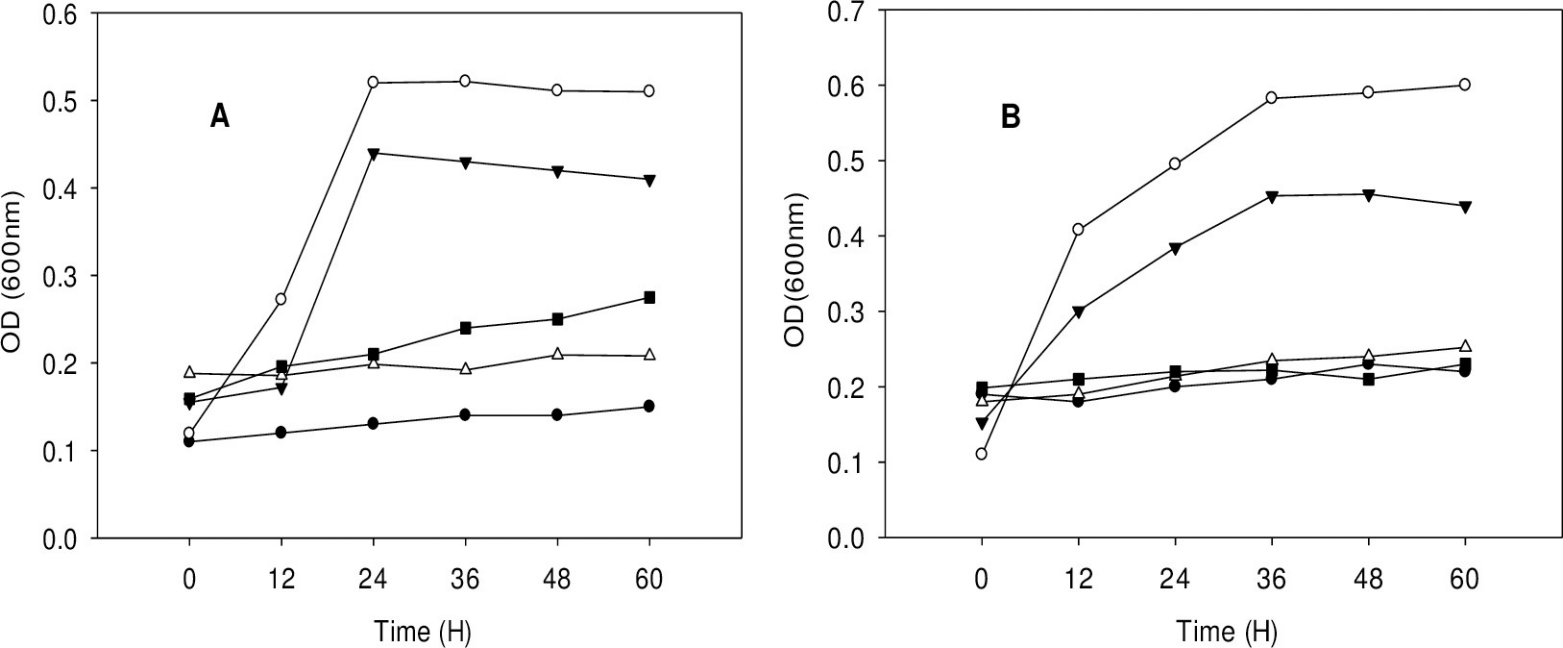
**Proliferation of the strains under different pH; (A) *Acinetobacter toweneri* NTA1-2A, (B) *Acinetobacter guillouiae* TAT1-6A.** Symbols: solid circle, pH 5; open circle, pH **6**; solid triangle, pH 7; open triangle, pH 8; solid square, pH 9 at 31°C and 1mM of 3MI concentration.

Bacterial growth was observed at temperatures of 25, 28, 31, 34 and 37°C and pH values of 5, 6, 7, 8, and 9 to determine optimal temperature and pH conditions. Both strains showed maximum growth without lag phase at a temperature of 31°C and pH 6 (Figs 3 and 4).

#### Effect of temperature

**Fig 5:**
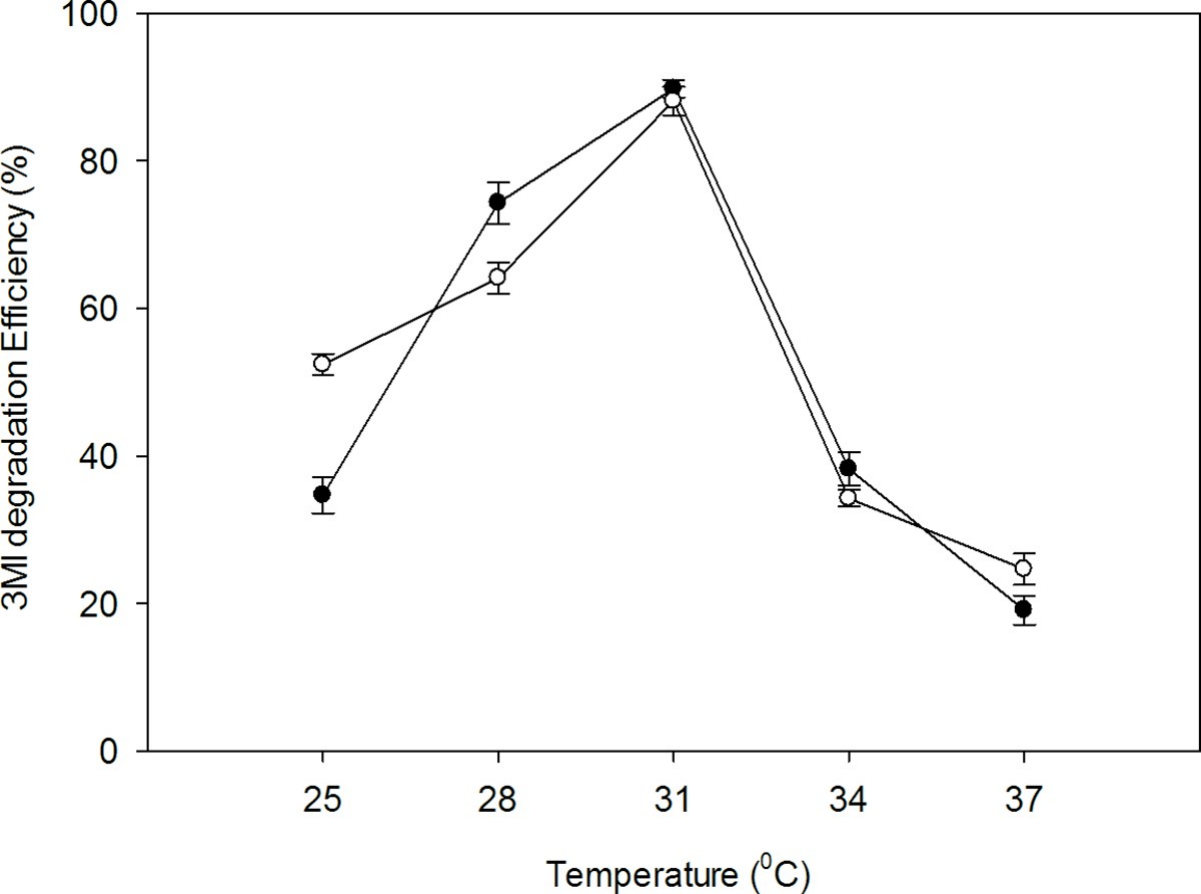
Effect of T (°C) on 3MI degradation efficiency by the strains at pH 6, and 1mM 3MI concentration. Symbols: open circle, NTA1-2A strain; solid circle, TAT1-6A strain.

The percent degradation efficiency of 3MI increased as the incubation temperature rose from 25°C to 31°C. Peak degradation efficiency was observed at 31°C and declined as temperature increased to 37°C (Fig 5).

#### Effect of pH

**Fig 6:**
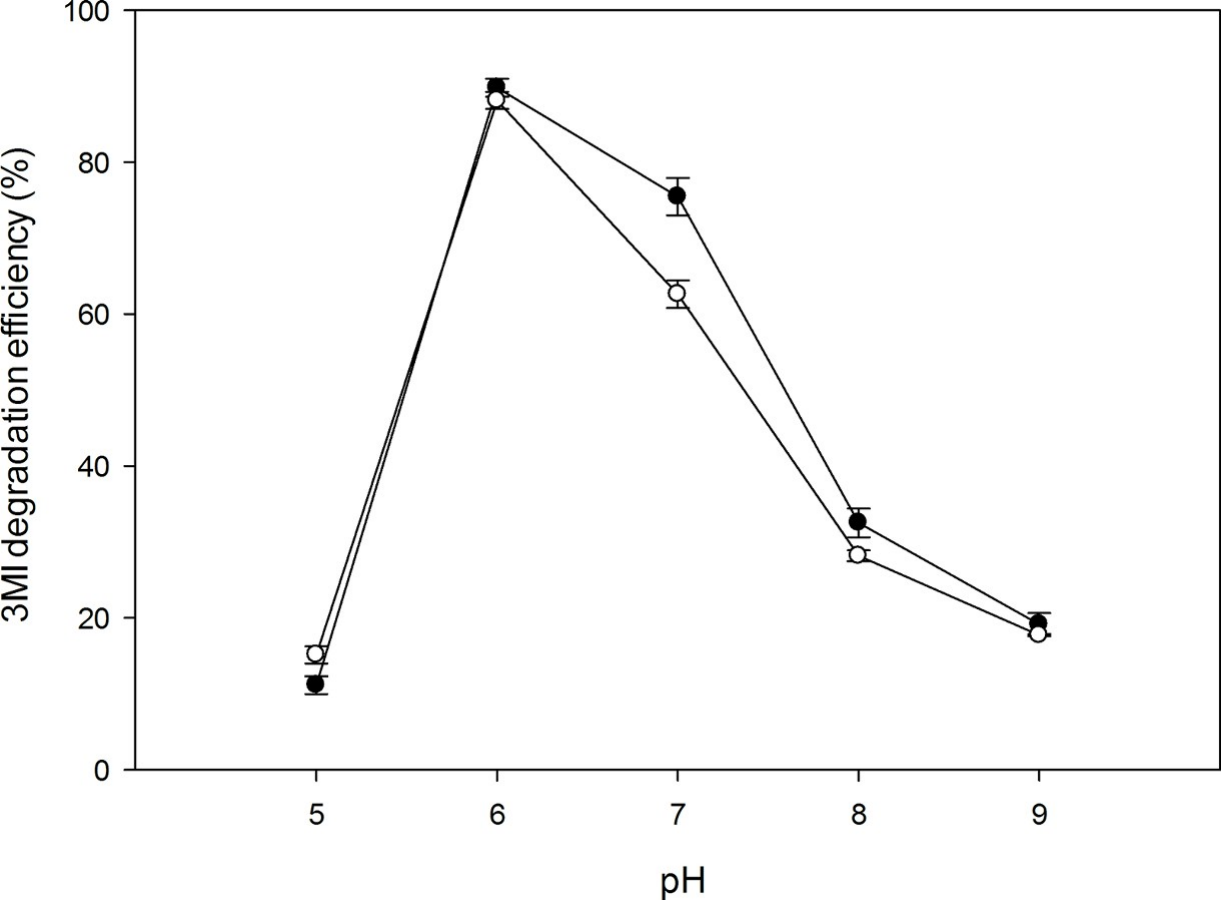
Effect of pH on 3MI degradation efficiency by the strains at T31, and 1 mM (131.17 mg/L) 3MI concentration. Symbols: open circle, NTA1-2A strain; solid circle, TAT1-6A strain.

3MI degradation using the two strains was also affected by the pH of culture media solutions. Both strains exhibited maximum 3MI degradation efficiency (%) at pH 6. Strain TAT1-6A showed increased 3MI degradation efficiency between pH 6 and 7 (Fig 6).

### 3MI degradation at different initial concentrations

In order to determine the percent degradation efficacy of the two strains, initial concentrations of 3MI ranging from 0.5 to 2.5 mM (65.58–327.92 mg/L) were inoculated with 100 μL of each strain and incubated for 6 d under aerobic conditions. Samples (2 mL) were collected every 24 h to determine 3MI concentration by HPLC. The results indicated that when the initial concentration of 3MI was 65.58 mg/L, the 3MI degradation efficiency was 100% and 98.95% with in 4 and 6 d by NTA1-2A and TAT1-6A strains respectively (Table 3). When the concentration of 3MI was 131.17 mg/L, the degradation efficiency was 98.67% for strain NTA1-2A and 97.99% for strain TAT1-6A over 6 d.

**Table 3:** Effect of initial concentration of 3MI on degradation ability of NTA1-2A and TAT1-6A strains.

		3MI degradation efficiency (%)	
Initial concentration (mg/L)	Incubation Time (h)	NTA1-2A	TAT1-6A
65.58	24	44.40±1.98^a^	23.34±1.84^b^
	48	69.94±1.53^a^	57.08±1.55^b^
	72	97.18±0.92^a^	73.60±0.46^b^
	96	100.00±0.0^a^	95.17±0.99^b^
	120	100.00±0.0^a^	97.91±1.15^b^
	144	100.00±0.0^a^	98.95±0.30^b^
131.17	24	20.50±1.23^a^	31.08±1.51^b^
	48	55.53±0.23	61.68±1.87
	72	76.44±1.43^a^	64.57±1.53^b^
	96	88.13±1.08	89.80±1.19
	120	98.00±0.68	97.50±0.67
	144	98.67±0.581	97.99±1.47
196.75	24	12.79±1.22^a^	20.44±1.30^b^
	48	29.11±1.31^a^	43.46±1.20^b^
	72	57.77±1.70^a^	51.88±1.80^b^
	96	67.86±1.88	71.28±1.03
	120	77.91±1.90^a^	83.66±1.15^b^
	144	85.98±1.78^a^	91.52±1.62^b^
262.34	24	6.75±1.64^a^	3.01±1.08^b^
	48	11.93±1.24	11.03±1.97
	72	17.45±1.25	14.68±1.24
	96	24.60±1.50^a^	17.22±0.41^b^
	120	26.28±1.12^a^	19.78±0.67^b^
	144	29.33±1.22^a^	23.67±0.44^b^
327.92	24	0.41±0.15	0.77±0.24
	48	4.33±0.21	1.03±0.31
	72	7.41±0.68^a^	2.21±0.18^b^
	96	8.04±0.69^a^	3.61±0.58^b^
	120	8.12±0.35^a^	4.11±0.47^b^
	144	8.18±0.44^a^	4.49±0.66^b^

Values are the mean ± standard error of triplicate assays.

^a, b^ means values with different letters in a row are significantly different (*P* < 0.05).

**Fig 7:**
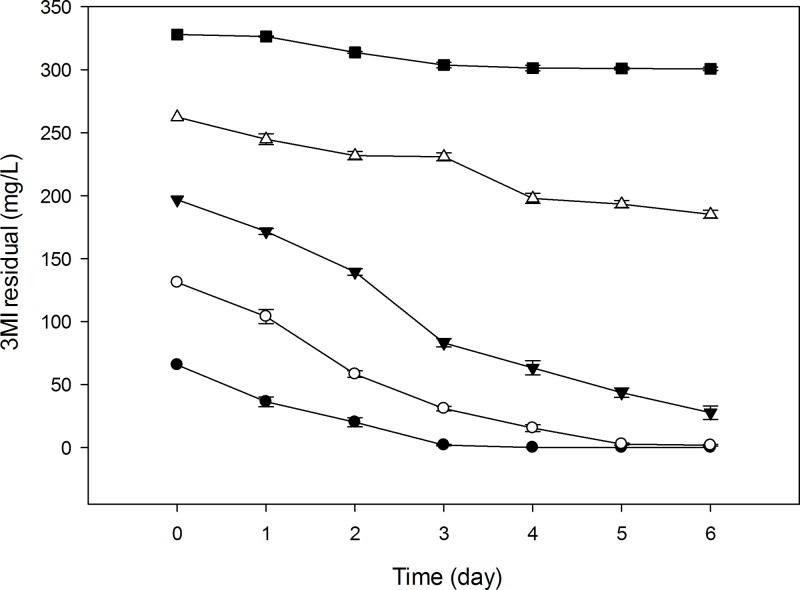
3MI removal capability of NTA1-2A strain at different initial concentrations (mg/L). Symbols: solid circle, 65.58; open circle, 131.17; solid triangle, 196.75; open triangle, 262.34; solid square, 327.92; Error bars, mean ±SE of three riplicates; at T = 31°C and pH 6.

**Fig 8:**
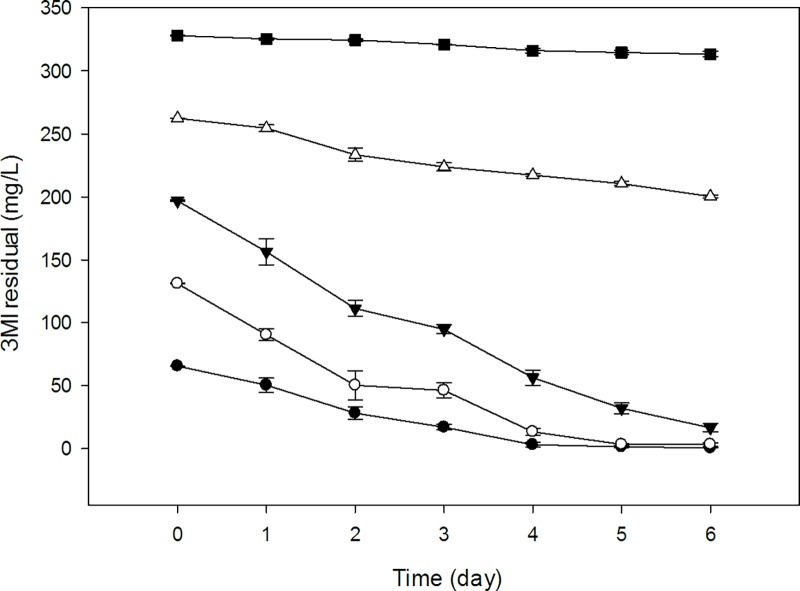
3MI removal capability of TAT1-6A strain at different initial concentrations (mg/L). Symbols: solid circle, 65.58; open circle, 131.17; solid triangle, 196.75; open triangle, 262.34; solid square, 327.92; Error bars, mean ±SE of three riplicates; at T = 31°C and pH 6.

The strains exhibited statistically significant differences in degradation efficiency (*P <* 0.05) for all starting 3MI concentrations, except at 131.17 mg/L (*P >* 0.05). In both strains, degradation efficiency decreased as 3MI concentration increased. Changes in 3MI degradation efficiency at different starting concentrations are illustrated by Figs 7 and 8.

### Enzyme activity

**Fig 9:**
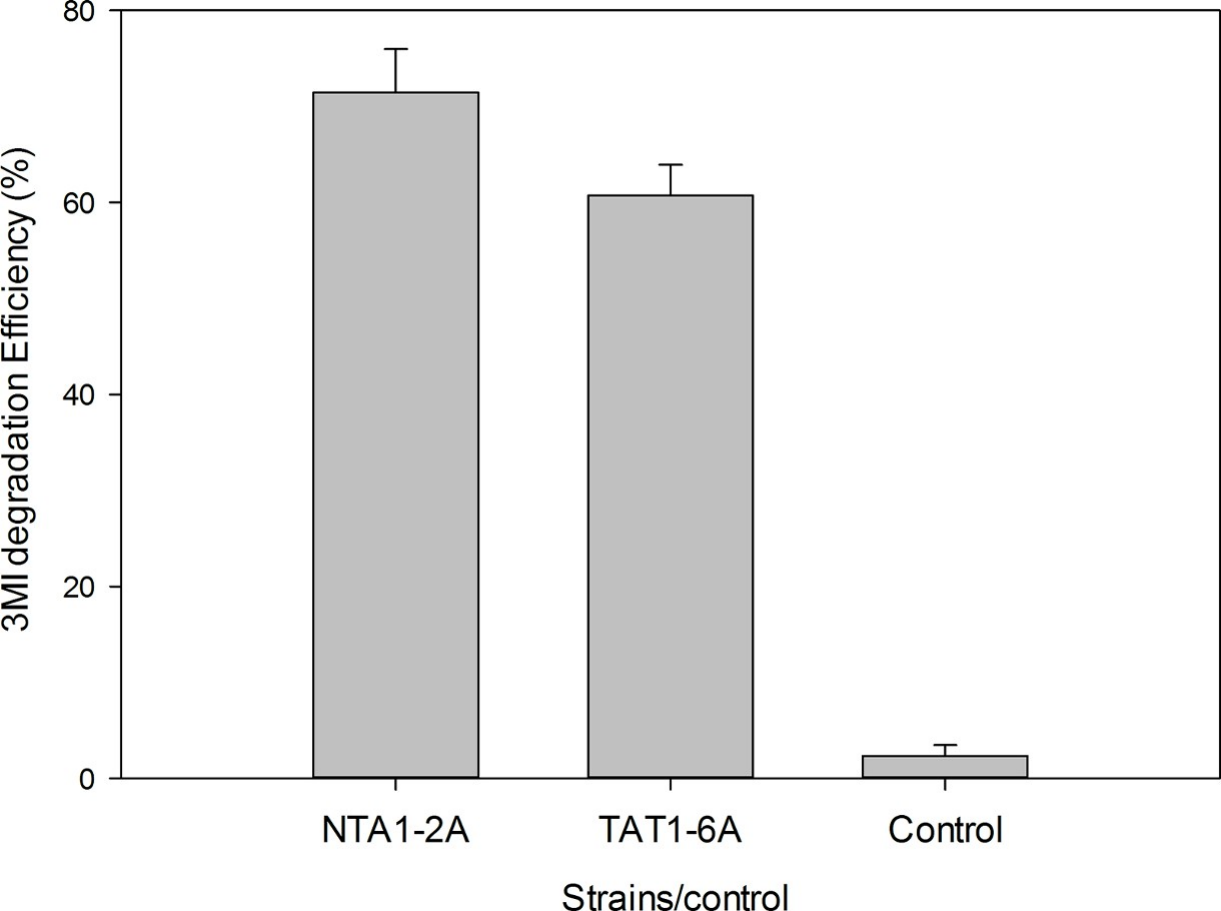
3MI degradation test by extract obtained from medium cultures of NTA1-2A and TAT1-6A. Error bars: mean ± SE of three replicates.

The enzyme activities of the crude extracts of cultures of the two strains (NTA1-2A and TAT1-6A) were measured (Fig 9). A concentration of 65.58 mg/L 3MI was used to test 3MI degradation activity. Culture extracts of NTA1-2A and TAT1-6A from the 3MI medium degraded 71.46% and 60.71% of 3MI as substrate, respectively. In the control group, there was no appreciable change in 3MI concentration. The results directly indicate that these strains have the ability to degrade 3MI.

### 3MI degradation metabolites

3-methylindole was degraded in the culture media inoculated with NTA1-2A and TAT1-6A strains when 3MI used as source of carbon and energy and degradation metabolites were identified. According to the information generated by LC-MS/MS^2^, the 3MI degradation metabolites were proposed to be C_5_H_11_NO_2_ (Betaine) and C_5_H_13_NO (Choline) by both strains. C_4_H_8_N_2_O (N-Nitroso-pyrrolidine) and C_6_H_13_NO_2_ (Beta-Alaninebetaine) were formed by NTA1-2A and TAT1-6A strains respectively.

### 3MI removal from chicken manure

Bacterial strains NTA1-2A and TAT1-6A were used to ferment chicken manure. The pretreatment 3MI content of the manure (0.86 ± 0.78 mg/kg) was used as a reference to determine removal efficiency of the strains. Removal efficacy over 8 d of 3-methylindole from the manure was 84.32% for strain NTA1-2A and 81.39% for strain TAT1-6A (Table 4). The removal efficiency of the strains showed a statistically-significant difference (*P <* 0.05) compared with the control group, but not between the two strains and their mix.

**Table 4:** 3MI removal efficiency of NTA1-2A and TAT1-6A from chicken manure. Values are mean ± SE of triplicate assays.

		3MI removal rate from chicken manure (%)		
Time (d)	NTA1-2A	TAT1-6A	Mix	Control
2	62.03±15.10^a^	81.99±4.54^b^	76.86±10.82^c^	1.32±0.01^d^
4	90.19±3.89^a^	84.45±5.88^a^	90.53±7.71^a^	43.01±1.15^b^
6	81.98±9.86^a^	82.96±8.64^a^	91.3±8.66^a^	31.34±1.38^b^
8	84.32±5.40^a^	81.39±5.34^a^	83.24±6.88^a^	41.32±1.910^b^

^a, b,c,d^ means values with different letters in a row show the strains are significantly different (P < 0.05) in their ability to remove 3MI from manure

## Discussion

Screening indigenous microorganisms is an ideal approach to find effective methods to biodegrade pollutants. In the last two decades, many pollutant-degrading microbes have been isolated from the environments, including strains capable of heterotrophic ammonia removal [28] and degradation of long-chain hydrocarbons [17] and aromatic compounds such as phenol and chlorophenol [19–21]. Because 3-methylindole is hazardous to the environment and human and animal welfare, and is difficult to degrade in nature, numerous studies have isolated and evaluated bacterial strains capable of degrading 3MI from different environments [9, 13–16, 28–31]. Our paper is the first report that the genus *Acinetobacter* isolated from chicken manure is capable of degrading 3MI. It is well-known that *Acinetobacter* are ubiquitous and important soil organisms, where they contribute to the mineralization of different organic compounds. They are strictly aerobic, with oxygen as terminal electron acceptor. Along with our results, these studies imply that *Acinetobacter spp*. may be promising candidates for pollution abatement in livestock husbandry and other industries, such as the petrochemical industry.

Among these 3MI-degrading bacterial strains, there is varying potential for and efficacy at removing 3MI. For instance, *Clostridium* species from pig and chicken manure treatment plant degraded 3MI at concentrations ranging from 100 to 300 mg/L in four weeks [2], while *Pseudomonas aeruginosa* Gs, isolated from mangrove sediment, degraded 3.0 mM (262.34 mg/L) of 3MI in 8 days [12, 22]. 3MI degradation using a methanogenic consortium derived from the enrichment of wetland soil converting 0.3 mM of 3MI to 3-methyloxindole after a 100 h lag period was reported [13]. The transformation of 3MI by methanogenic-bacterial consortia from marine sediment was also investigated in Victoria Harbor [15], where a sulfate-reducing consortium mineralized 0.4 mM of 3MI completely in 35 d [31]. The removal of 3MI was also studied in four strains of lactic acid bacteria (*Lactobacillus brevis* 1.12 (*L*. *brevis* 1.12), *L*. *plantarum* 102, *L*. *casei* 6103, and *L*. *plantarum* ATCC8014) in which the 3MI removal ability of *L*. *brevis* 1.12 was the strongest among the four strains, and the highest removal rate was 65.35% in 1 mL incubation medium containing 1.0 mg/L 3MI for 120 h [14]. Sharma *et al*. [16] isolated a new 3MI-degrading purple non-sulfur bacterium, *Rhodopseudomonas palustris* WKU-KDNS3, from a swine waste lagoon in which the bacterium removed >93% of 90 μM 3MI supplied to the medium in 21 days. Aerobic biodegradation and comprehensive biotransformation mechanisms of 3MI revealed in a soil bacterium *Cupriavidus sp*. Strain KK10 which biodegraded 100 mg/L of 3MI in 24 hours [9]. Compared with the above species, our strains NTA1-2A and TAT1-6A exhibited rapid degradation efficiency at concentrations ranging from 65–200 mg/L within the first 6 days (Figs 7 and 8). Additionally, the degradation potential of the two strains isolated in this study is better compared with others in 3MI removal ability except *Cupriavidus sp*. Strain KK10 [9].

Apart from the degradation potential and efficiency, tolerance of strains to 3MI and its metabolites is critical because 3MI is mildly toxic and widely resistant to degradation [29]. For example, *Pseudomonas aeruginosa* Gs isolated from mangrove sediment tolerated initial concentrations up to 3.0 mM (393.51 mg/L) [12, 22], but at concentrations greater than 3.0 mM the growth of the strain was inhibited. *Clostridium malenominatum* A-3 could degrade 3MI under anaerobic conditions, and the maximum growth allowance concentrations were 100–300 mg/L [2]. Our strains NTA1-2A and TAT1-6A can tolerate 3MI initial concentrations up to 200 mg/L with >85% degradation efficiency, when the 3MI initial concentration was 262.34 mg/L 6 days degradation efficiency was >23% (Table 3), but degradation was very slow and strain growth was inhibited. This is consistent with a study by Yin and Gu [22] that reported higher concentrations of 3MI inhibited biodegradation and growth of bacteria.

Bacterial degradation of 3MI resulted different metabolites in previous studies. For instance, metabolism of 3-methylindole via methyloxindole was reported in methanogenic consurtum [13]. Gu et al [15] proposed 3MI degradation via 3-methyloxindole and α-methyl-2-aminobenzencacetic acid by marine anaerobic microorganisms. Two metabolites of 3MI degradation were detected and proposed to be indoline-3-carboxylic acid and indoline-3-ol by *Pseudomonas aeruginosa* Gs [22]. Cometabolic 3-methylindole biodegradation was also confirmed biotransformation products in which carbocyclic aromatic ring-fission of 3-methylindole to single-ring pyrrole carboxylic acids [9]. Some researchers reported 3-methylindole as biotransformation metabolite of indole acetic acid [11, 32–35]. Anaerobic bacteria such as Lactobacillus sp.[32] and Clostridium species[33] transformed indole acetic acid to 3-methylindole. Fukoka et al [36] also reported biotransformation of indole in *Cupriavidus sp*.strain KK10 through N-hetrocyclic or carboxylic aromatic ring cleavage. Recently, 3-methylindole also reported as a biotransformation product of indole without ring cleavage in *Lysinibacillus xylanilyticus* strain MA [37]. In our experiment, C_5_H_11_NO_2_ (Betaine) and C_5_H_13_NO (Choline) identified by both strains while C_4_H_8_N_2_O (N-Nitroso-pyrrolidine) and C_6_H_13_NO_2_ (Beta-Alaninebetaine) were formed by NTA1-2A and TAT1-6A strains respectively. The metabolites detected in our present study were not reported in previous studies. These metabolites are most probably formed by ring cleavage of 3MI by enzymatic activity of the strains.

Bacterial degradation of 3MI is affected by several different factors such as temperature, pH, and salinity [22, 38, 39]. Temperature and pH of the culture medium greatly influence bacterial growth and 3MI-degradation rate by affecting the activity of various microbial enzymes. One study reported temperature and pH as major factors affecting indole biodegradation (Madsen et al [40]), while another also indicated that the degradation rate of poly-aromatic hydrocarbons was influenced by these factors (Sihag et al [41]). In this study, 3MI degradation efficiency was affected by temperature and pH for strains NTA1-2A and TATT1-6A (Figs 5 & 6). The strains degraded 3MI over a wide range of temperatures from 25 to 37°C and pH 6 to 7 with maximum degradation efficiency at 31°C and pH 6. These temperature and pH conditions are very similar to the early stage of compositing manure mixing reported in other studies [42, 43].

Bacterial isolates degrade organic pollutants via production of intracellular or extracellular enzymes. In our two strains, the degradation of 3MI by initial enrichment cultures was confirmed by observing decreasing 3MI concentrations in MSM and growth of bacterial strains in MSM. Degradation of *n*-alkane by *Acinetobacter* sp. is coordinated by a dioxygenase enzyme [17], and methyl oxidation of 3MI by cytochrome P450 enzymes [44]. It was reported that enzyme extract or supernatant fluid from the culture media of 3MI-degrading isolates showed degradation following incubation with 3MI [3, 14]. In our study, the successful degradation of 3MI by the supernatant of the fluid media from the two strains was observed after incubation for 48 h (Fig 9). The supernatant fluid of the culture media of the two strains also had the ability to remove 3MI, with removal efficiencies of 71.46% and 60.71% in 48 hours. Similar results was reported using lactic acid bacterium *L*. *brevis* 1.12 [14]. Strains NTA1-2A and TAT1-6A were also tested *in vivo* during fermentation of chicken manure. The two strains and the mix (NTA1-2A and TAT1-6A) showed significant differences in the ability to remove 3MI from chicken manure, and all presented good prospects for application in the biodegradation of nitrogenous organic pollutants (such as 3MI) in poultry and other livestock industries.

## Conclusions

This study is the first to identify 3MI degradation in *Acinetobacter* species. Two *Acinetobacter* strains capable of utilizing 3MI were isolated from chicken manure and identified as *Acinetobacter towneri* NTA1-2A and *Acinetobacter guillouiae* TAT1-6A based on 16S rDNA gene amplicon sequence analysis. The optimum conditions for the two strains are pH 6 and 31°C with 3MI as source of carbon. The strains can degrade initial concentration of 3MI ranging from 65 to 200 mg/L. The degradation efficiency is >85% in 6 days in both strains when the initial concentration is less than 200 mg/L. Our experiments revealed that betaine and choline were identified as 3MI degradation metabolites by both strains while nitroso-pyrrolidine and beta-alaninebetaine formed by NTA1-2A and TAT1-6A strains respectively. NTA1-2A and TAT1-6A have exhibited the capability of removing 3MI from chicken manure. We conclude that the two strains have the potential to minimize noxious fecal odorants due to indolic compounds in the poultry and other livestock industries. Hence, future studies should focus on the application of strains, and investigate the biochemical and genetic basis of degradation of 3MI by the two strains.

## Supporting information

S1 Table 3-methylindole (3MI) residual (mg/Kg) detected by HPLC during degradation by different strains isolated from chicken manure. (DOCX)

S2 Table **Proliferation of the strains under different temperature;** (A) ***Acinetobacter toweneri* NTA1-2A, (B) *Acinetobacter guillouiae* TAT1-6A using 3-methylindole (131.17 mg/L) as source of carbon.**
(DOCX)

S3 Table **Proliferation of the strains under different pH;** (**A) Acinetobacter toweneri NTA1-2A, (B) Acinetobacter guillouiae TAT1-6A using 3-methylindole (131.17 mg/L) as source of carbon.**
(DOCX)

S4 Table Effect of T ^0^ C on 3-methylindole degradation efficiency (%) by the strains at pH6, and 1 mM (131.17 mg/L) 3-methylindole concentration. (DOCX)

S5 Table Effect of pH on 3-methylindole degradation efficiency (%) by the strains NTA1-2A and TAT1-6A at T31, and 1 mM (131.17 mg/L). (DOCX)

S6 Table 3-methylindole residual detected during degradation by NTA1-2A strain at different initial concentrations (mg/L). (DOCX)

S7 Table 3-methylindole reidual detected during degradation by TAT1-6A strain at different initial concentrations (mg/L). (DOCX)

S8 Table 3MI degradation test by supernatant fluid or enzyme extract obtained from media cultures of NTA1-2A and TAT1-6A. (DOCX)
