# Supplementary material for: Isolation and characterization of two Acinetobacter species able to degrade 3-methylindole
Source: PLoS One. 2019 Jan 28;14(1):e0211275. doi: 10.1371/journal.pone.0211275 (PMC6349333; doi:10.1371/journal.pone.0211275)
Supplement: S1 Table — (DOCX) [file pone.0211275.s001.docx]

**S1 Table. 3-methylindole (3MI) residual (mg/Kg) detected by HPLC during degradation by different strains isolated from chicken manure.**

| Strains | S1 | S2 | S3 | Average |
| --- | --- | --- | --- | --- |
| TAT1-6A | 55.27 | 45.23 | 50.31 | 50.27 |
| NTA1-2A | 32.35 | 38.63 | 38.40 | 36.46 |
| 6B | 64.21 | 64.69 | 64.93 | 64.61 |
| 2B | 61.54 | 63.64 | 63.23 | 62.81 |

***Initial 3MI concentration was 65.58mg/L**
