# Supplementary material for: Isolation and characterization of two Acinetobacter species able to degrade 3-methylindole
Source: PLoS One. 2019 Jan 28;14(1):e0211275. doi: 10.1371/journal.pone.0211275 (PMC6349333; doi:10.1371/journal.pone.0211275)
Supplement: S2 Table — Proliferation of the strains under different temperature; (A) Acinetobacter toweneri NTA1-2A, (B) Acinetobacter guillouiae TAT1-6A using 3-methylindole (131.17 mg/L) as source of carbon. (DOCX) [file pone.0211275.s002.docx]

**S2 Table. Proliferation of the strains under different temperature; (A) *Acinetobacter toweneri* NTA1-2A, (B) *Acinetobacter guillouiae* TAT1-6A using 3-methylindole as source of carbon*.***

|  |  | Absorbance (OD 600nm) of culture media at different temperature | | | | |
| --- | --- | --- | --- | --- | --- | --- |
| Strains | Time (H) | OD  (25 ^0^C) | OD  (28 ^0^C) | OD  (31 ^0^C) | OD  (34 ^0^C) | OD (37 ^0^C) |
| NTA1-2A | 0 | 0.15 | 0.03 | 0.03 | 0.03 | 0.03 |
|  | 12 | 0.14 | 0.05 | 0.43 | 0.12 | 0.18 |
|  | 24 | 0.16 | 0.78 | 1.12 | 0.88 | 0.32 |
|  | 36 | 0.4 | 1.13 | 1.19 | 0.62 | 0.48 |
|  | 48 | 0.43 | 1.17 | 1.18 | 0.5 | 0.43 |
|  | 60 | 0.48 | 1.10 | 1.15 | 0.35 | 0.40 |
| TAT1-6A | 0 | 0.15 | 0.07 | 0.03 | 0.03 | 0.03 |
|  | 12 | 0.18 | 0.16 | 0.40 | 0.19 | 0.03 |
|  | 24 | 0.34 | 1.19 | 1.24 | 0.38 | 0.03 |
|  | 36 | 0.59 | 1.20 | 1.23 | 0.60 | 0.06 |
|  | 48 | 0.72 | 1.24 | 1.20 | 0.52 | 0.27 |
|  | 60 | 0.67 | 1.22 | 1.19 | 0.45 | 0.46 |
