# Supplementary material for: Isolation and characterization of two Acinetobacter species able to degrade 3-methylindole
Source: PLoS One. 2019 Jan 28;14(1):e0211275. doi: 10.1371/journal.pone.0211275 (PMC6349333; doi:10.1371/journal.pone.0211275)
Supplement: S3 Table — Proliferation of the strains under different pH; (A) Acinetobacter toweneri NTA1-2A, (B) Acinetobacter guillouiae TAT1-6A using 3-methylindole (131.17 mg/L) as source of carbon. (DOCX) [file pone.0211275.s003.docx]

**S3 Table. Proliferation of the strains under different pH; (A) Acinetobacter toweneri NTA1-2A, (B) *Acinetobacter guillouiae* TAT1-6A using 3-methylindole (131.17 mg/L) as source of carbon*.***

|  |  | Absorbance (OD) ( 600 nm) of culture media at different pH | | | | | |
| --- | --- | --- | --- | --- | --- | --- | --- |
| Strains | Time (H) | OD (pH 5) | OD (pH 6) | OD (pH 7) | OD (pH 8) | OD (pH 9) | |
| NTA1-2A | 0 | 0.11 | 0.12 | 0.16 | 0.19 | 0.16 | |
|  | 12 | 0.12 | 0.27 | 0.17 | 0.19 | 0.20 | |
|  | 24 | 0.13 | 0.52 | 0.44 | 0.20 | 0.21 | |
|  | 36 | 0.14 | 0.52 | 0.43 | 0.19 | 0.24 | |
|  | 48 | 0.14 | 0.51 | 0.42 | 0.21 | 0.25 | |
|  | 60 | 0.15 | 0.51 | 0.41 | 0.21 | 0.28 | |
| NTA1-2A | 0 | 0.19 | 0.11 | 0.15 | 0.24 | 0.28 |  |
|  | 12 | 0.18 | 0.41 | 0.30 | 0.25 | 0.28 |  |
|  | 24 | 0.20 | 0.50 | 0.39 | 0.31 | 0.30 |  |
|  | 36 | 0.21 | 0.58 | 0.45 | 0.35 | 0.32 |  |
|  | 48 | 0.23 | 0.67 | 0.46 | 0.39 | 0.30 |  |
|  | 60 | 0.22 | 0.62 | 0.44 | 0.38 | 0.29 |  |
