# Supplementary material for: Isolation and characterization of two Acinetobacter species able to degrade 3-methylindole
Source: PLoS One. 2019 Jan 28;14(1):e0211275. doi: 10.1371/journal.pone.0211275 (PMC6349333; doi:10.1371/journal.pone.0211275)
Supplement: S4 Table — (DOCX) [file pone.0211275.s004.docx]

**S4 Table. Effect of T ^0^ C on 3-methylindole degradation efficiency (%) by the strains at pH6, and 1 mM (131.17 mg/L) 3-methylindole concentration.**

| Temperature (^0^C) | 3MI by NTA1-2A (%) | 3MI by TAT1-6A (%) |
| --- | --- | --- |
| 25 | 52.41 | 34.67 |
| 28 | 64.17 | 74.34 |
| 31 | 88.13 | 89.80 |
| 34 | 34.24 | 38.25 |
| 37 | 24.65 | 19.10 |

*Note the data are mean of triplicate samples
