# Supplementary material for: Isolation and characterization of two Acinetobacter species able to degrade 3-methylindole
Source: PLoS One. 2019 Jan 28;14(1):e0211275. doi: 10.1371/journal.pone.0211275 (PMC6349333; doi:10.1371/journal.pone.0211275)
Supplement: S5 Table — (DOCX) [file pone.0211275.s005.docx]

**S5 Table. Effect of pH on 3MI degradation efficiency (%) by the strains NTA1-2A and TAT1-6A at T31, and 1 mM (131.17 mg/L).**

| pH | 3MI by NTA1-2A (%) | 3MI by TAT1-6A (%) |
| --- | --- | --- |
| 5 | 15.12 | 11.12 |
| 6 | 88.13 | 89.80 |
| 7 | 64.62 | 66.47 |
| 8 | 28.15 | 28.49 |
| 9 | 17.7 | 19.14 |

**Note the data are mean of triplicate samples
