# Supplementary material for: Isolation and characterization of two Acinetobacter species able to degrade 3-methylindole
Source: PLoS One. 2019 Jan 28;14(1):e0211275. doi: 10.1371/journal.pone.0211275 (PMC6349333; doi:10.1371/journal.pone.0211275)
Supplement: S6 Table — (DOCX) [file pone.0211275.s006.docx]

**S6 Table. 3-methylindole residual detected during degradation by NTA1-2A strain at different initial concentrations (mg/L)**

|  | 3MI detected per 24hr by strain NTA1-2A | | | |
| --- | --- | --- | --- | --- |
| 3MI Initial concentration (mg/l) | Time | S1 | S2 | S3 |
| 65.58 | 24 | 36.86 | 32.35 | 40.17 |
|  | 48 | 19.02 | 16.48 | 23.63 |
|  | 72 | 1.84 | 1.25 | 2.45 |
|  | 96 | 0.00 | 0.00 | 0.00 |
|  | 120 | 0.00 | 0.00 | 0.00 |
|  | 144 | 0.00 | 0.00 | 0.00 |
| 131.17 | 24 | 104.95 | 98.42 | 109.46 |
|  | 48 | 58.58 | 55.56 | 60.86 |
|  | 72 | 31.00 | 28.98 | 32.74 |
|  | 96 | 15.92 | 12.67 | 18.10 |
|  | 120 | 2.40 | 1.86 | 3.61 |
|  | 144 | 1.73 | 2.51 | 0.99 |
| 196.75 | 24 | 171.50 | 174.02 | 169.23 |
|  | 48 | 139.48 | 142.04 | 136.89 |
|  | 72 | 83.13 | 79.73 | 86.44 |
|  | 96 | 63.29 | 57.54 | 68.87 |
|  | 120 | 43.42 | 39.75 | 47.24 |
|  | 144 | 27.60 | 22.10 | 33.05 |
| 262.34 | 24 | 244.42 | 240.44 | 249.02 |
|  | 48 | 231.35 | 227.65 | 234.14 |
|  | 72 | 216.46 | 210.73 | 222.52 |
|  | 96 | 197.74 | 193.92 | 201.77 |
|  | 120 | 193.62 | 190.35 | 196.23 |
|  | 144 | 185.74 | 182.03 | 188.42 |
| 327.93 | 24 | 326.94 | 326.04 | 326.78 |
|  | 48 | 313.80 | 314.34 | 312.98 |
|  | 72 | 303.66 | 301.40 | 305.83 |
|  | 96 | 302.02 | 299.12 | 303.56 |
|  | 120 | 301.94 | 299.99 | 301.99 |
|  | 144 | 301.95 | 299.40 | 301.94 |
