# Supplementary material for: Isolation and characterization of two Acinetobacter species able to degrade 3-methylindole
Source: PLoS One. 2019 Jan 28;14(1):e0211275. doi: 10.1371/journal.pone.0211275 (PMC6349333; doi:10.1371/journal.pone.0211275)
Supplement: S7 Table — (DOCX) [file pone.0211275.s007.docx]

**S7 Table. 3-methylindole reidual detected during degradation by TAT1-6A strain at different initial concentrations (mg/L)**

|  | 3MI detected per 24hr by strain TAT1-6A | | | |
| --- | --- | --- | --- | --- |
| 3MI Initial concentration (mg/L) | Time (H) | S1 | S2 | S3 |
| 65.58 | 24 | 50.01 | 56.2 | 44.62 |
|  | 48 | 28.07 | 33.14 | 23.24 |
|  | 72 | 17.89 | 19.23 | 14.81 |
|  | 96 | 3.16 | 5.13 | 1.21 |
|  | 120 | 1.32 | 2.15 | 0.64 |
|  | 144 | 0.91 | 0.53 | 0.62 |
| 131.17 | 24 | 90.40 | 85.81 | 95.01 |
|  | 48 | 50.27 | 61.89 | 38.62 |
|  | 72 | 46.80 | 52.23 | 40.37 |
|  | 96 | 13.21 | 16.32 | 10.58 |
|  | 120 | 3.20 | 2.451 | 4.194 |
|  | 144 | 1.11 | 2.004 | 4.813 |
| 196.75 | 24 | 156.81 | 145.97 | 166.81 |
|  | 48 | 111.17 | 117.56 | 104.98 |
|  | 72 | 94.63 | 98.24 | 91.15 |
|  | 96 | 56.75 | 50.43 | 62.34 |
|  | 120 | 32.45 | 27.78 | 36.21 |
|  | 144 | 16.82 | 19.80 | 13.41 |
| 262.34 | 24 | 254.37 | 251.65 | 257.31 |
|  | 48 | 233.44 | 228.23 | 238.54 |
|  | 72 | 223.91 | 227.04 | 220.54 |
|  | 96 | 217.14 | 218.24 | 216.10 |
|  | 120 | 210.45 | 208.70 | 212.23 |
|  | 144 | 200.23 | 201.42 | 199.10 |
| 327.93 | 24 | 325.52 | 326.10 | 324.54 |
|  | 48 | 324.78 | 325.46 | 323.43 |
|  | 72 | 320.12 | 320.56 | 321.32 |
|  | 96 | 316.08 | 318.00 | 314.20 |
|  | 120 | 314.40 | 316.02 | 312.91 |
|  | 144 | 313.02 | 315.43 | 311.12 |
