# Supplementary material for: Isolation and characterization of two Acinetobacter species able to degrade 3-methylindole
Source: PLoS One. 2019 Jan 28;14(1):e0211275. doi: 10.1371/journal.pone.0211275 (PMC6349333; doi:10.1371/journal.pone.0211275)
Supplement: S8 Table — (DOCX) [file pone.0211275.s008.docx]

**S8 Table. 3MI degradation test by supernatant fluid or enzyme extract obtained from media cultures of NTA1-2A and TAT1-6A.**

| code | 3MI degradation by NTA1-2A | 3MI degradation by TAT1-6A | control |
| --- | --- | --- | --- |
| S1 | 19.05 | 25.49 | 62.89 |
| S2 | 16.13 | 22.50 | 62.89 |
| S3 | 20.47 | 28.61 | 64.58 |
|  | Note: The 3MI concentration is the amount detected (mg/Kg) from the culture media 65mg/L of 3MI used as initial cononcentration. | | |
